# A rare primary tumor of the thyroid gland: report a new case of leiomyosarcoma and literature review

**DOI:** 10.1186/1746-1596-8-36

**Published:** 2013-02-27

**Authors:** Bennani Amal, Hinde El Fatemi, Ihsane Souaf, Kaouthar Moumna, Amarti Affaf

**Affiliations:** 1 Departement of pathology, Hassan II University Hospital, Fez 30000, Morocco

**Keywords:** Primary leiomyosarcoma, Thyroid gland, Anaplastic carcinoma, Medullary carcinoma

## Abstract

Primary leiomyosarcomas of the thyroid gland are extremely rare. we report a case of a 72 year-old women with a painful growing mass of the left neck with skin fistula. The patient underwent a lobectomy. The tumor histology showed spindle-shaped cells arranged in interlacing fascicles that expressed desmine and Hcaldesmone, but were negative for cytokeratins and thyroglobulin. Total body CT scan didnt show any other tumor. The patient died two months after surgery. Primary thyroid leiomyosarcoma may be mistaken for other tumors, such as anaplastic or medullary carcinomas. Therefore, the diagnosis is difficult and requires numerous clinical, radiological, and pathological investigations.

**Virtual Slides:**

The virtual slide(s) for this article can be found here: http://www.diagnosticpathology.diagnomx.eu/vs/1917621950869224

## Introduction

Leiomyosarcomas are commonly seen in the pelvis, the gastrointestinal tract and the retroperitoneum. They account for 6% of the head and neck tumors. Sarcomas are thought to originate from the wall of blood vessels [1]. Primary leiomyosarcomas of the thyroid gland are extremely rare. To the best of our knowledge, only 18 cases have been reported around the world. Immunohistochemistry showing a smooth muscle-specific antigen is helpful in establishing the diagnosis. Surgical excision is the primary treatment of choice. Survival rate is low and approximately half of the patients die within a short period of time after the diagnosis. We report this new case in the aim of shedding more light on the primary leiomyosarcoma of the thyroid and the challenges in making the diagnosis and the differential diagnosis with anaplastic and medullary carcinomas.

## Case report

We present a case of a 72 year-old woman with a rapidly growing neck mass over a period of two months. Her past medical history includes a multinodular goiter diagnosed two years ago with no follow-up.

At the physical exam we found a painful mass of the left neck with fistulae to the skin. She is clinically euthyroid with normal plasma level of thyroid stimulating hormones. A total body scan didn’t show any other mass.

The patient underwent a surgical exploration. There was a hard, irregular tumor of the whole left lobe of the thyroid gland, which was attached to the sterno-thyroidian muscle and the left cervical pedicle. A left lobectomy was realized. As the tumor largely invaded the surrounding tissues, a curative operation seemed impossible.

**Figure 1:**
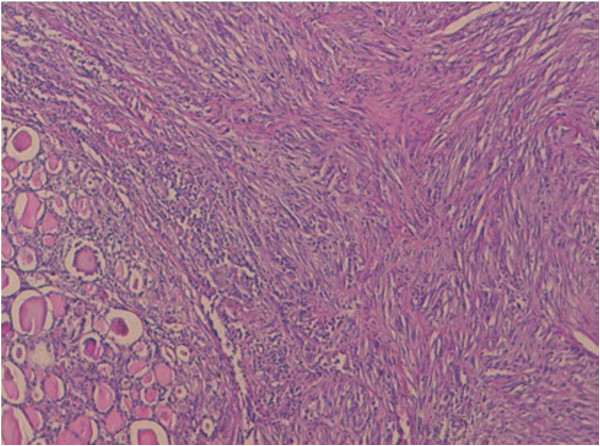
The tumor is compactly cellular and is composed of spindle-shaped (hematoxylin-eosin-safran × 10).

**Figure 2:**
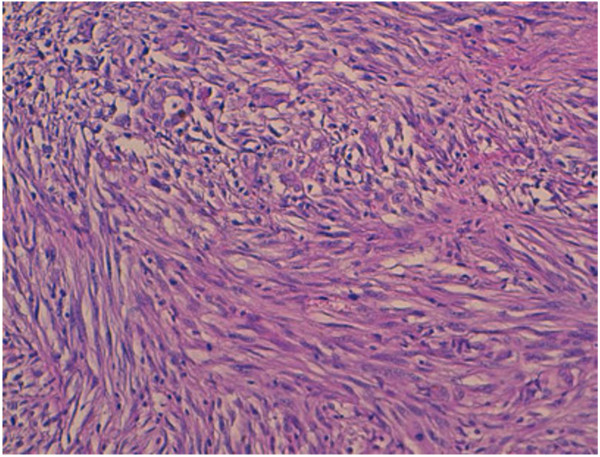
Nuclear cigarshaped nuclei with pleomorphism and mitosis, (hematoxylin-eosin-safran × 40).

The resected lobe measured 8.5 cm in the largest dimension; there is a multinodular appearance with a yellowish nodule measuring 5 cm in diameter. On histology, the lesion was focally well defined, but unencapsulated and was composed of interlacing fascicles of atypical spindle and epithelioid cells (Figure 1). These cells had an abundant eosinophilic cytoplasm and a hyperchromatic blunt-ended and cigar shaped nuclei with marked anisocaryosis and high mitotic ratio (Figure 2). The stroma was scant, essentially composed of capillaries with rare lymphocytes. Some areas of necrosis were found.

**Figure 3:**
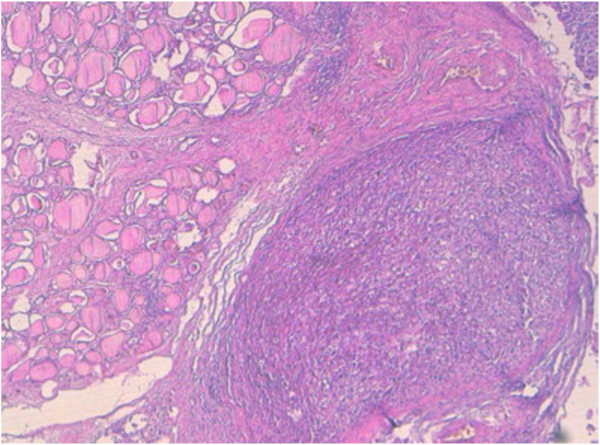
The tumor often invades the surrounding thyroidian parenchyma with some non tumoral thyroid follicles trapped into the tumor (×5).

The tumor invaded the surrounding thyroidian parenchyma with some non tumoral thyroid follicles trapped into the tumor (Figure 3).

**Figure 4:**
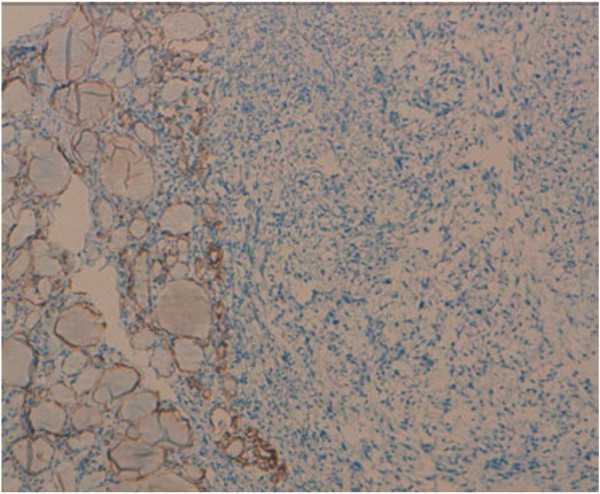
Immunohistochemical study with cytokeratin which was negative.

**Figure 5:**
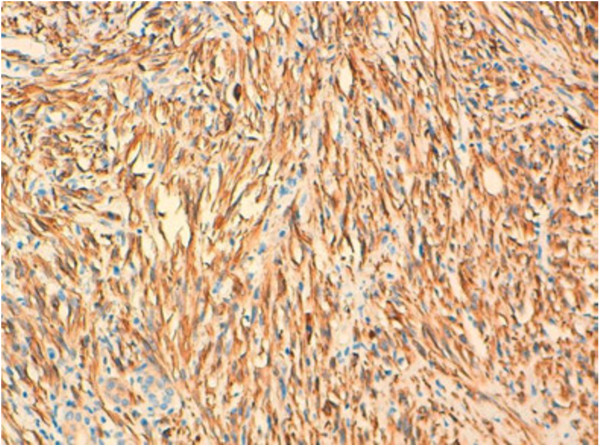
The tumor cells are stained with anti–hcaldesmone.

**Figure 6:**
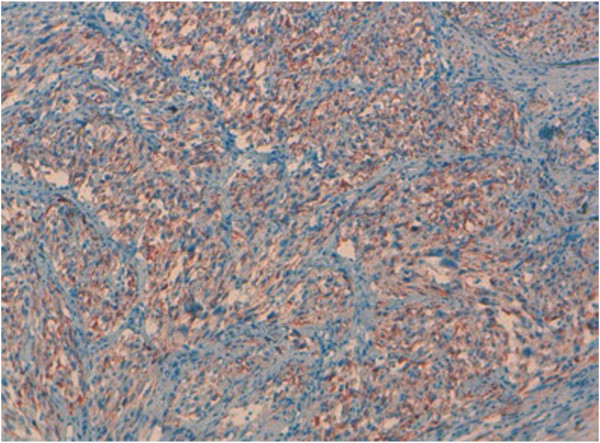
The tumor cells are stained with anti–desmine.

**Figure 7:**
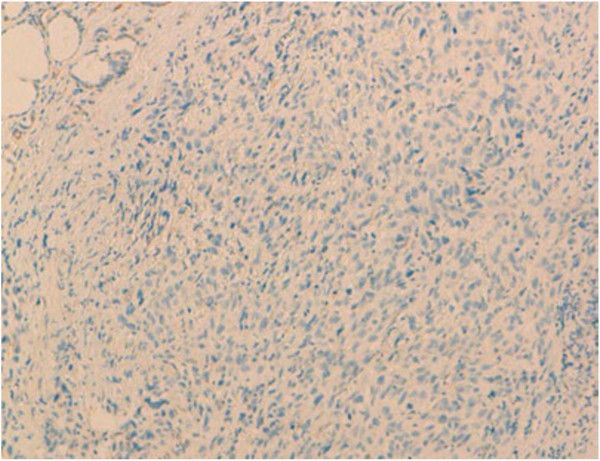
There is no positivity for estrogen receptor.

**Figure 8:**
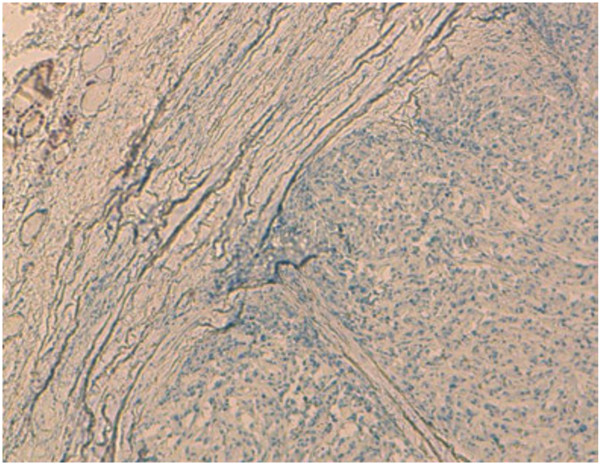
There is no positivity for TTF1.

**Figure 9:**
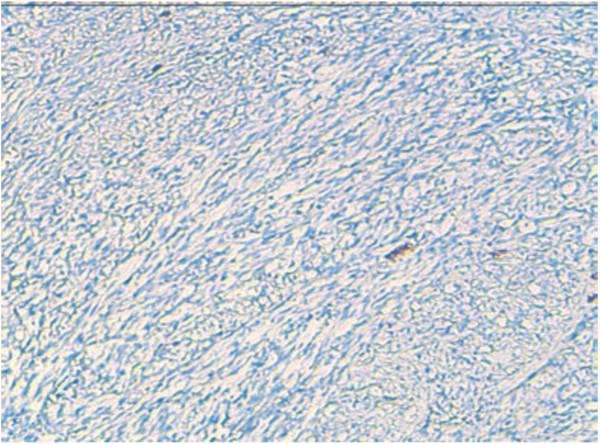
There is no positivity for chromogranine or synaptophysine.

Undifferentiated thyroid carcinoma was suspected. The immunohistochemical study with cytokeratin was negative (Figure 4). Secondary immunohistochemical stains showed positive diffusion for Hcaldesmone (Figure 5), and focal positivity for desmine ( Figure 6) in the spindle cells all the other stains performed (chromogranine, synaptophysine, TTF1, estrogen receptor) were negative (Figure 7, Figure 8, Figure 9).

## Discussion

Thyroid tumors are the most common endocrine tumors in the United States, and about 40% of the population between 30 and 60 years-old have thyroid nodules, most of which are benign [2]. A survey sponsored by the World Health Organization (WHO) in 2010 revealed that there are around 44,670 new cases and 1,690 deaths caused by Thyroid carcinoma every year [3]. Papillary carcinoma of the thyroid (PTC) is the commonest thyroid cancer [4], while follicular thyroid carcinoma (FTC) accounts for 10 -17% of clinically evident thyroid malignancies [5].

Primary leiomyosarcoma of the thyroid gland is exceedingly rare and accounts for 0.014% of 28 630 thyroid tumors in one series [6]. It occurred in older patients with a mean age of 67 years with a predominance of women, though one pediatric case of a 6-year-old boy had been reported [7]. Patients often complained of an enlarging cervical mass with Obstructive symptoms.

Most reports in the literature showed that the thyroid function test is often normal. Thyroid scans can show a cold nodule or an enlarged gland with areas of increased and decreased uptake of radioactive iodine. Ultrasounds show an ill-defined or well-defined hypoechogenic mass, a solid partially cystic nodule , or a calcified nodule. At computed tomography, the tumor presents as a low-density mass with dense calcification and necrotic portions. In addition, computed tomography sometimes shows a direct tumoral invasion of the adjacent structures. In the current case, the patient had clinically a locoregional extension.

At gross examination, the tumors either almost entirely replace one thyroid lobe or presents as an irregularly outlined or a well-demarcated intrathyroidian nodule.

Cut surfaces show a pinkish, yellowish, white, fascicular or homogenous, unencapsulated nodule with necrosis or cystic degeneration. Histologicaly, the tumor is composed of interlacing fascicles or bundles of eosinophilic spindle cells with normochromatic to hyperchromatic, blunt-ended, and cigar shaped nuclei, generally centrally located within the cell. Mitoses are occasional to frequent. Variable levels of cellular and nuclear pleomorphism are present. Areas of hemorrhage, hyalinization, myxoid changes, and necrosis may be found with occasional calcifications.The tumor often invades the surrounding thyroidian parenchyma. Vascular invasion can be seen. Tumor cells are stained with vimentin, (alpha-) smooth muscle actin, and muscle-specific actin with a variable expression of desmin. Cytokeratin, thyroglobulin, calcitonin, protein S100, and chromogranin are never expressed.

The diagnosis of primary leiomyosarcomas of the thyroid gland requires particular prudence, because they are often ill-defined, and invade the thyroid capsule and the adjacent cervical structures. It seems important to exclude a direct extension to the thyroid gland by a non thyroidian cervical leiomyosarcoma. Indeed, approximately 1% of the head and neck sarcomas are leiomyosarcomas, most commonly located at the scalp, paranasal sinuses, and jaws [8]. Surgical explorations as well as gross and microscopic pathologic examinations are indispensable for a correct identification of tumor origin. In our case, it showed a tumor of the thyroid that was attached to the muscle and cervical pedicle. Furthermore, it is important to exclude thyroid metastases of a distant leiomyosarcoma. It is believed that approximately 1% of thyroid cancers are metastases to the thyroid gland. In autopsy series, thyroid metastases have been found to occur in up to 24% of patients who died of cancer [9]. Thus, only clinical examination and appropriate imaging studies will help in making the diagnosis. In our case, both physical exam and total body scan didn’t show any other tumor. Moreover, more features were in favor of primitive lesion: it is a solitary lesion (all the few cases of metastatic leiomyosarcoma reported in literature presented clinically with multiple metastasis at lungs and bones), and it had bad outcome (patient died within two months after surgery).

The main differential pathologic diagnoses includes undifferentiated (anaplastic) thyroid carcinomas (UTCs), solitary fibrous tumors, spindle cell tumors with thymus-like differentiation, medullary carcinoma and other sarcomas. Undifferentiated (anaplastic) thyroid carcinomas may display a very polymorphic microscopic features, including spindle cells that can mimic the microscopic appearance of fibrosarcoma, leiomyosarcoma, or malignant fibrous histiocytoma. Epithelial markers can be missing in approximately 20% of cases [10] and even more often in spindle cell sarcomatoid UTCs. Vimentin is also expressed in more than 50% of UTCs [11]. Desmin and muscle-specific actin, which are good markers of leiomyosarcomas, are never expressed in UTCs [11]. Solitary fibrous tumors are predominantly composed of spindle cells, with various arrangement. It is usually positive for CD34, BCL2, CD99, and vimentin. Spindle cell tumors with thymus-like differentiation is in most case biphasic, composed of compact interlacing, or reticulated highly cellular fascicles of spindle cells and tubulopapillary glands. In immunohistochemistry, both spindle cells and glandular cells are positive for cytokeratins, and rarely the spindle cells exhibit staining with some muscle markers. Medullary carcinoma can mimic any tumor structurally and functionally [12]. It can show variable microscopic features, including spindle cells. In immunochemistry, tumor cells are positive for chromogranine A, synaptophysine, ACE and calcitonine.

The etiology of primary leiomyosarcomas of the thyroid gland still remains unknown. No history of previous cervical radiation exposure in such cases had been reported till now. As it was observed that a blood vessel was the point of origin of a leiomyosarcoma of the thyroid gland [13], the histogenesis of leiomyosarcoma may be from the smooth muscle in the vascular walls.

In our case, the diagnosis of leiomyosarcoma was made on the pathological, and immunohistochemical features of the tumor, which were similar to those found in the literature.

Despite surgical excision, most patients die after 1 to 51 months or get a metastatic disease: (lungs, lymph nodes, liver, myocardium, kidney, pancreas, small bowel, colon, peritoneum, brain, and bones) [14]. Two cases have been reported, in which the patients were still alive with no evidence of disease, with a follow-up of 15 and 25 months [15].

## Conclusion

In summary, primary leiomyosarcoma of the thyroid is extremely rare. It is a challenging diagnosis to make due to its differential diagnosis with anaplastic and medullary carcinoma. But before making this diagnosis, it is important to exclude a metastasis to the thyroid gland of a distant leiomyosarcoma or a thyroid extension of a non thyroidian cervical leiomyosarcoma.

## Consent

Written informed consent was obtained from the patient’s parents for publication of this case report and any accompanying images.

## Competing interests

The authors declare that they have no competing interests.

## Authors’ contributions

AB wrote the manuscript and performed the literature review. HE revised the manuscript for important intellectual content, IS, KM and AA were major contributors to writing the manuscript. All authors read and approved the final manuscript.
